# Reintervention in Artificial Cardiac Pacing Systems

**DOI:** 10.5935/abc.20180222

**Published:** 2018-11

**Authors:** Silas dos Santos Galvão Filho

**Affiliations:** Centro Avançado de Ritmologia e Eletrofisiologia (CARE), São Paulo, SP - Brazil

**Keywords:** Electrodes, Implanted, Intraoperative Complications, Catheter Ablation, Pacemaker, Artificial/trends, Arrhythmias, Cardiac

Reintervention in artificial cardiac pacing systems that involves lead approach, either for the implantation of a new lead and/or the removal of an old one, is most frequently a difficult procedure, with a high probability of complications. Since the transvenous endocardial route started being used by artificial cardiac pacing systems, lead cables have shown to be more vulnerable to complications;^1^ however, when it comes to reintervention, the complications are much more frequent.

The significant prevalence of venous obstruction^2^ and the consequent difficulty in obtaining a new venous access, the complexity of percutaneous extraction of old lead cables,^3^ in addition to the higher prevalence of surgical infections,^4^ constitute some of the complications that determine the greater complexity of reinterventions.

The study “Usefulness of preoperative venography in patients with cardiac implantable electronic devices submitted to lead replacement or device upgrade procedures”,^5^ calls attention to this ever-growing problem,^6^ as the implantable electronic cardiac devices use more lead cables and increase the patients’ life expectancy, in addition to emphasizing the importance of a previous venography to program the approach strategy. In the present study, approximately 1/4 of the patients submitted to reintervention had severe venous obstructions or occlusions. In such cases, when a new lead cable is required, the extraction of old ones may be absolutely necessary to attain access.

The venous system exploration through the venography can be performed intraoperatively; however, prior knowledge of possible obstructions allows better programming of the surgical procedure, with a previous request of special materials, such as mechanical or laser-energized sheath systems for lead cable extraction, which should always be available in these cases.

Moreover, considering the cost of these special materials, it is very important in the real world and in our country to have prior authorization from the health care providers to use them, determining cost predictability and minimizing problems when charging for the procedure. The agreement between the programmed and the actual surgical procedure, which occurred in the study in 99% of the cases, strongly reinforced the importance of performing a prior venography when scheduling reintervention procedures.

The lack of knowledge of venous obstructions at the reinterventions leads to the unavailability of lead extraction systems during the procedure, and in those cases requiring the implantation of new lead(s) and in which access cannot be attained, implantation of a contralateral artificial cardiac pacing system while abandoning the old lead cables may be the only option. However, the increase in surgical time, which can result in a higher risk of infection, as well as the increased number of implanted leads, are considerable drawbacks of this approach.

Advances in technology with the development of leadless pacemaker systems will, in the future, address problems with transvenous leads. Nevertheless, the current state of this technology^7^^,^^8^ with the use of single-chamber devices, is still not able in most cases, to dispense with traditional dual-chamber artificial heart pacing systems with leads, suggesting we will be facing such situations for a long time yet.

As they constitute one of the most difficult and delicate surgical procedures in the area of artificial heart stimulation, re-interventions in lead cables must be very well programmed, in addition to requiring a level of high expertise by the surgeon/rhythmologist. In this sense, performing a venography prior to the procedure is very important, as it was well demonstrated by this article.
